# Vanishing Gastroschisis: The Importance of Prenatal Diagnosis in a Seemingly Normal Abdomen

**DOI:** 10.1055/a-2692-6661

**Published:** 2025-09-12

**Authors:** Clara Massaguer, Irene De Haro Jorge, Laura Saura García, Jordi Prat Ortells, María Elena Muñoz Fernández, Xavier Tarrado

**Affiliations:** 1 Department of Pediatric Surgery, Hospital Sant Joan de Déu, Esplugues de Llobregat, Barcelona, Spain; 2 Department of Pediatric Surgery, Consorci Corporació Sanitària Parc Taulí, Sabadell, Spain

**Keywords:** vanishing gastroschisis, gastroschisis, intestinal failure, short bowel syndrome, serial transverse enteroplasty

## Abstract

A newborn of 32 + 6 weeks' gestational age with prenatal diagnosis of gastroschisis was born through elective caesarean section. Ultrasonography at 16 + 4 gestational weeks (GW) showed a gastroschisis with free bowel loops floating in amniotic fluid. From 27 + 4 GW onward, serial ultrasounds showed the disappearance of extra-abdominal intestine and progressive intra-abdominal intestinal loops dilation, raising suspicion for vanishing gastroschisis. Birth weight was 2,136 grams and the external appearance of the abdomen was normal. An exploratory laparotomy was performed, finding a dilated proximal jejunal loop with a type III intestinal atresia, microcolon, and no other remainder bowel in between. The total length of the small intestine was 21 cm. Serial transverse enteroplasties for intestinal lengthening (reaching 38 cm), along with lateroterminal jejunocolic anastomosis were performed. The patient was discharged after 5 months of hospitalization with home parenteral nutrition. At 2 years and 8 months of age, the child is thriving and off parenteral support.

Vanishing gastroschisis is a rare and severe form of complex gastroschisis whose prenatal diagnosis is crucial for parental counseling, timely delivery, and early surgical intervention. Multidisciplinary approach is essential to manage intestinal failure and improve long-term outcomes in these patients.

## Introduction

Vanishing gastroschisis is a rare condition in which the abdominal wall defect spontaneously closes in utero, leading to the loss of extra-abdominal bowel and typically resulting in intestinal atresia and short bowel syndrome (SBS). 1 Compared with other forms of complex gastroschisis, intra-abdominal bowel dilation tends to occur earlier during prenatal follow-up. 2 Early prenatal ultrasound signs that should raise suspicion for closing and vanishing gastroschisis include a small abdominal wall defect combined with absence of extra-abdominal bowel dilation. 3 4 5 Recognizing these subtle imaging clues is crucial for providing accurate prenatal counseling to parents, especially regarding the potential risk of intestinal failure and the long-term quality of life compromise of their child. 6 If not diagnosed prenatally, the abdominal wall may appear normal at birth, potentially delaying diagnosis and increasing the risk of preventable complications, such as bowel perforation. 7

In cases of SBS, maximizing bowel preservation is critical due to the limited remaining length. Intestinal lengthening techniques, such as the serial transverse enteroplasties (STEP) procedure, are often employed to enhance bowel function. 8 9 10 We present a case of vanishing gastroschisis with secondary very short bowel managed with a primary STEP procedure.

## Case Report

**Fig. 1:**
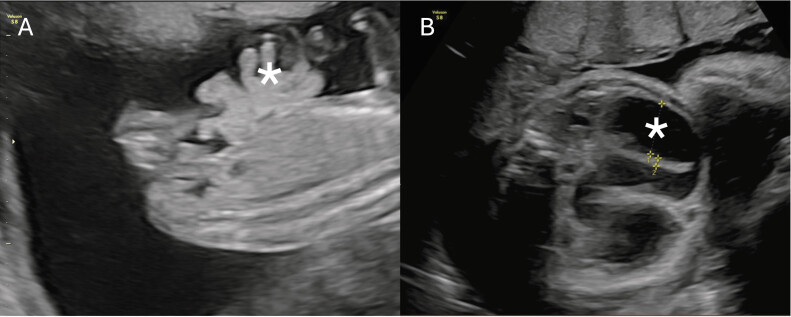
( **A** ) Ultrasound at 16 + 4 weeks of gestation showing free extra-abdominal intestinal loops, diagnostic of gastroschisis. *: extra-abdominal bowel loops. ( **B** ) Ultrasound at 32 + 3 weeks of gestation. No extra-abdominal intestinal loops are identified. Intra-abdominal loops are dilated up to 25 to 28 mm, greater than previous ultrasounds, with increased peristalsis. No ascites or hydrops signs were observed. Fetal Doppler and growth were normal. *: intestinal loops.

**Fig. 2:**
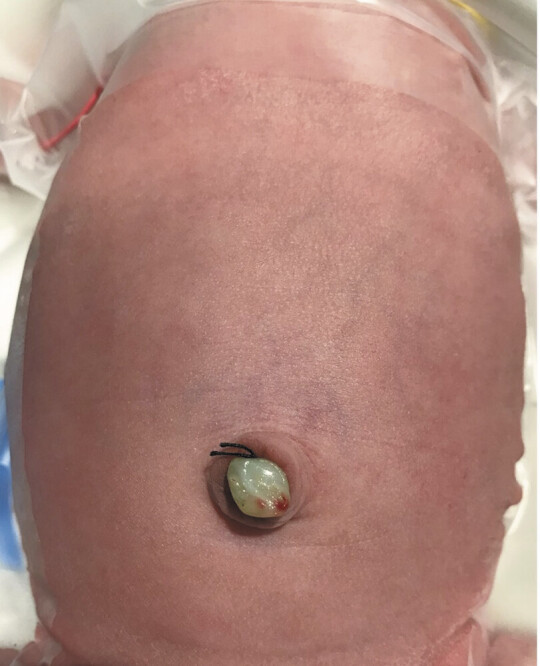
Clinical exam at birth. Abdomen appearance was normal without any stigma of abdominal wall defect.

A male infant with prenatal diagnosis of gastroschisis was born at 32 + 6 weeks' gestational age via elective caesarean section. At 16 + 4 weeks, ultrasonography revealed free bowel loops in amniotic fluid ( Fig. 1A ). Amniocentesis and fetal array were negative for genetic abnormalities. Ultrasound controls were performed without significant changes. However, ultrasound at 27 + 4 weeks showed the disappearance of extra-abdominal bowel and, in successive exams, progressive dilation of intra-abdominal loops with increased peristalsis was observed, suggesting a vanishing gastroschisis ( Fig. 1B ). Due to progressive intra-abdominal bowel dilation (up to 28 mm), early delivery was scheduled at 32 + 6 weeks to prevent intrauterine complications. The infant weighed 2,136 grams at birth and the abdomen looked normal ( Fig. 2 ).

**Fig. 3:**
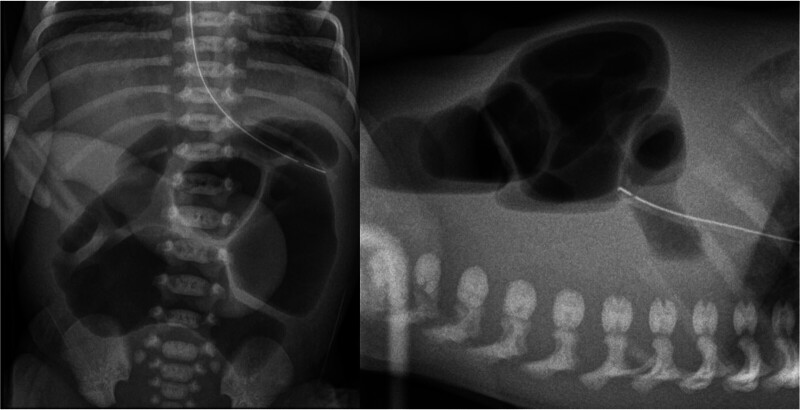
Abdominal X-ray with dilated bowel and air–fluid levels.

**Fig. 4:**
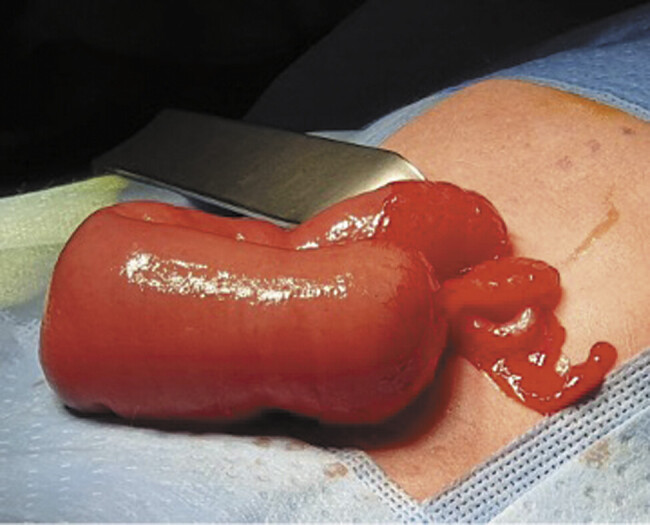
Surgical findings: Type IIIa jejunal atresia, absence of the remaining small bowel, and microcolon.

Abdominal X-ray displayed a dilated bowel with air–fluid levels and abdominal ultrasound showed a fixed thickened central bowel loop ( Fig. 3 ). Exploratory laparotomy confirmed a dilated proximal jejunal loop with type IIIa intestinal atresia, absence of the remaining small bowel, and half colon and microcolon from transverse portion on ( Fig. 4 ). Only 21 cm of small bowel remained, including the duodenum. STEP procedure was done, lengthening the intestine up to 38 cm, followed by a lateroterminal jejunocolic anastomosis.

Parenteral nutrition was initiated on day 2 and trophic feeding with maternal milk was started on day 11. Enteral volume was very gradually increased due to frequent vomiting and malabsorption. The infant remained in the neonatal intensive care unit for 63 days and was discharged at 5 months on home parenteral nutrition, which was gradually reduced over time.

He experienced multiple complications with his permanent tunneled central catheter, including rupture requiring repair and two replacements (one due to thrombosis causing malfunction and the other due to persistent symptomatic granuloma with cuff extrusion). He also had three central-line-associated bacteremias and cholestasis due to long-term parenteral nutrition. The central catheter was removed at 2 years and 6 months.

At 2 years and 8 months, he is properly thriving and off parenteral nutrition. He weighs 12 kg, eats normally supplemented with nutritional shakes, and passes three stools per day.

## Discussion

Vanishing gastroschisis is a rare but severe variant of gastroschisis associated with a high risk of morbidity. 1 It often entails SBS, with significant implications for nutrition, growth, and quality of life. 6 7 Early prenatal detection is crucial and the hallmark clinical challenge is the often deceptively normal abdomen at birth, which may delay diagnosis. 4 5 In cases without prenatal diagnosis, the infant will present with obstructive symptoms. In such scenario, performing a contrast enema may be very useful for the differential diagnosis (e.g., showing a microcolon in cases of intestinal atresia or meconium ileus, a change of caliber in Hirschsprung's disease, or a stop in the pass of contrast suggestive of colonic atresia, among others). 11

Given the limited bowel length of our patient and the dilation due to the atresia, STEP procedure was performed at the time of the initial surgery. While STEP is typically reserved for the management of bowel dilation and adaptation over time, in selected cases like this, its early use can help increase absorptive surface area and slow transit, potentially improving enteral tolerance from the outset. 8 10 12

Despite appropriate medical and nutritional management, the patient faced multiple complications associated with long-term central venous access, including catheter-related infections, thrombosis, granuloma, and cholestasis secondary to long-term parenteral nutrition. 13 14 These are common complications in SBS patients and reflect the importance of multidisciplinary intestinal rehabilitation programs. 6 9

## Conclusion

This case underscores the importance of early prenatal recognition of vanishing gastroschisis findings, to enable proper prenatal diagnosis, parental counseling, and planning of the delivery at a specialized center. Primary bowel-lengthening techniques in cases of SBS associated with a very dilated bowel should be considered during initial repair. The successful weaning from parenteral nutrition in this case highlights the potential for intestinal adaptation, even in infants with ultrashort bowel length.

## Notes

Importance for the Pediatric Surgeon Vanishing gastroschisis is a rare but severe condition often resulting in significant bowel loss and short bowel syndrome whose early prenatal recognition is essential for timely intervention and improved outcomes. Primary STEP can be a valuable surgical option in cases with extreme intestinal shortening and significant bowel dilation. Multidisciplinary follow-up is key to achieving enteral autonomy and long-term growth.

## Notes

**Erratum:** This article was published with incorrect page numbers. The page numbers have been corrected subsequently with the publication of an erratum (DOI: 10.1055/a-2706-9632).

## References

[1] Raval M V Bensard D D Karrer F M Sato T T Vanishing gastroschisis: clinical and pathologic findings J Pediatr Surg 2008 43 10 1912 191518926232

[2] Feldkamp M L Carey J C Pimentel R Krikov S Botto L D Etiology and clinical presentation of gastroschisis: an epidemiologic study Birth Defects Res A Clin Mol Teratol 2007 79 07 525 530

[3] Ko H S Lee Y Kim H Y Park I Sohn Y S Prenatal sonographic findings of vanishing gastroschisis Ultrasound Obstet Gynecol 2008 31 06 699 701

[4] Mackenzie T C Harrison M R Farmer D L Gastroschisis: a clinical review Pediatr Surg Int 2004 20 05 319 32515185108

[5] Geslin D Clermidi P Gatibelza M E What prenatal ultrasound features are predictable of complex or vanishing gastroschisis? A retrospective study Prenat Diagn 2017 37 02 168 175 27981591 10.1002/pd.4984

[6] Modi B P Langer M Duggan C Improved survival in very short bowel syndrome: the role of parenteral nutrition and intestinal adaptation J Pediatr Surg 2006 41 05 992 997

[7] Puligandla P S Janvier A Flageole H Bouchard S Leclerc S Skarsgard E D Perinatal management of gastroschisis: a national survey J Pediatr Surg 2004 39 03 395 400

[8] Kim H B Fauza D Garza J Oh J T Nurko S Jaksic T Serial transverse enteroplasty (STEP): a novel bowel lengthening procedure J Pediatr Surg 2003 38 03 425 429 12632361 10.1053/jpsu.2003.50073

[9] Dariel A Faure A Martinez L European Pediatric Surgeon' Association survey on the management of short-bowel syndrome Eur J Pediatr Surg 2021 31 01 8 13 33197945 10.1055/s-0040-1721040

[10] Garnett G M Kang K H Jaksic T First STEPs: serial transverse enteroplasty as a primary procedure in neonates with congenital short bowel J Pediatr Surg 2014 49 01 104 107, discussion 108 24439591 10.1016/j.jpedsurg.2013.09.037

[11] Baad M Delgado J Dayneka J S Anupindi S A Reid J R Diagnostic performance and role of the contrast enema for low intestinal obstruction in neonates Pediatr Surg Int 2020 36 09 1093 1101 32572600 10.1007/s00383-020-04701-4

[12] Wales P W Christison-Lagay E R Short bowel syndrome: epidemiology and etiology Semin Pediatr Surg 2010 19 01 3 9 20123268 10.1053/j.sempedsurg.2009.11.001

[13] Venick R S Calkins K L Current management of intestinal failure in children Curr Opin Organ Transplant 2021 26 05 538 544

[14] Thompson J S Rochling F A Weseman R A Mercer D F Current management of short bowel syndrome Curr Probl Surg 2012 49 02 52 115 22244264 10.1067/j.cpsurg.2011.10.002

